# Association of Health Care Factors With Excess Deaths Not Assigned to COVID-19 in the US

**DOI:** 10.1001/jamanetworkopen.2021.25287

**Published:** 2021-09-13

**Authors:** Andrew C. Stokes, Dielle J. Lundberg, Jacob Bor, Irma T. Elo, Katherine Hempstead, Samuel H. Preston

**Affiliations:** 1 Department of Global Health, Boston University School of Public Health, Boston, Massachusetts; 2 Department of Epidemiology, Boston University School of Public Health, Boston, Massachusetts; 3 Department of Sociology and Population Studies Center, University of Pennsylvania, Philadelphia; 4 Robert Wood Johnson Foundation, Princeton, New Jersey

## Abstract

This cross-sectional study assesses health care factors associated with excess deaths not assigned to COVID-19 in US counties in 2020.

## Introduction

Approximately 20% of excess deaths in the US in 2020 were not reflected in COVID-19 death counts.^1,2,3^ These excess deaths included deaths caused by COVID-19 but not assigned to it as well as indirect deaths from other causes associated with delays in health care and the social and economic consequences of the pandemic. Prior research has documented differences in the percentage of excess deaths not assigned to COVID-19 at the state and county levels.^1,3,4^ In this study, we examined health care factors associated with excess deaths not assigned to COVID-19 at the county level.

## Methods

For this cross-sectional study, we used US National Center for Health Statistics data on deaths from COVID-19 and all-cause deaths occurring in US counties from January 1 to December 31, 2020. We also used the Centers for Disease Control and Prevention WONDER data on all-cause deaths from 2013 to 2018 and US Census Bureau population data. The present study relied on deidentified publicly available data and was therefore exempted from review and the requirement for informed consent by the Boston University Medical Center institutional review board. This study followed the Strengthening the Reporting of Observational Studies in Epidemiology (STROBE) reporting guideline.

We modeled all-cause mortality in 2020 as a function of historical all-cause mortality from 2013 to 2018 and directly assigned deaths from COVID-19 in 2020. The coefficient relating directly assigned deaths from COVID-19 to all-cause mortality was used to calculate the percentage of excess deaths not assigned to COVID-19. Next, we stratified our model by health care factors (eMethods in the Supplement). This analysis was conducted using Stata, version 16 (StataCorp).

## Results

**Figure 1.**
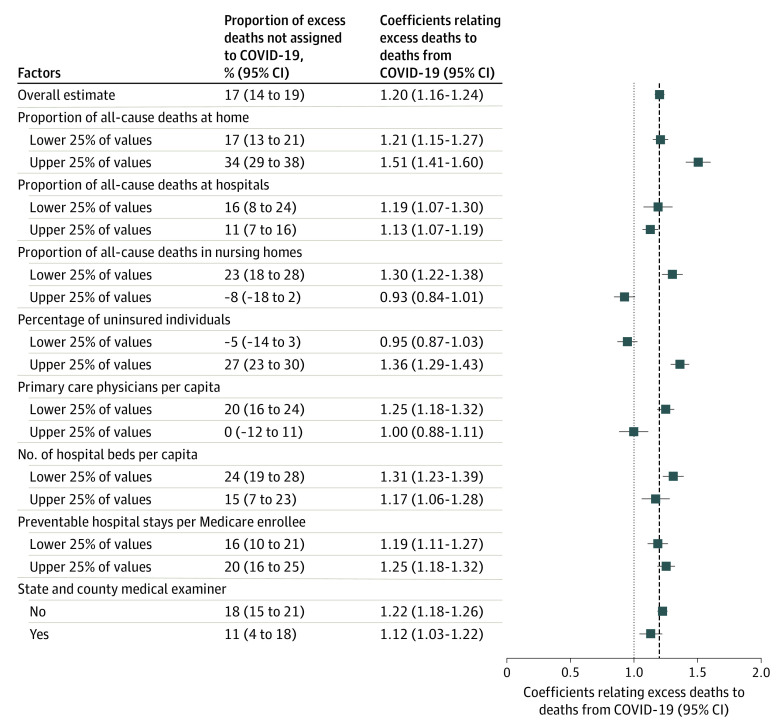
Percentage of Excess Deaths Not Assigned to COVID-19 in 2096 US Counties in 2020, by Health Care Factors Coefficients were generated according to the model given in the eMethods in the Supplement. The model was weighted by the 2020 population and fully stratified by health system factors. For continuous measures, factors were divided into population-weighted quartiles. The coefficients relating excess deaths to deaths from COVID-19 can be interpreted as the number of excess deaths that occurred for every 1 directly assigned death from COVID-19.

**Figure 2.**
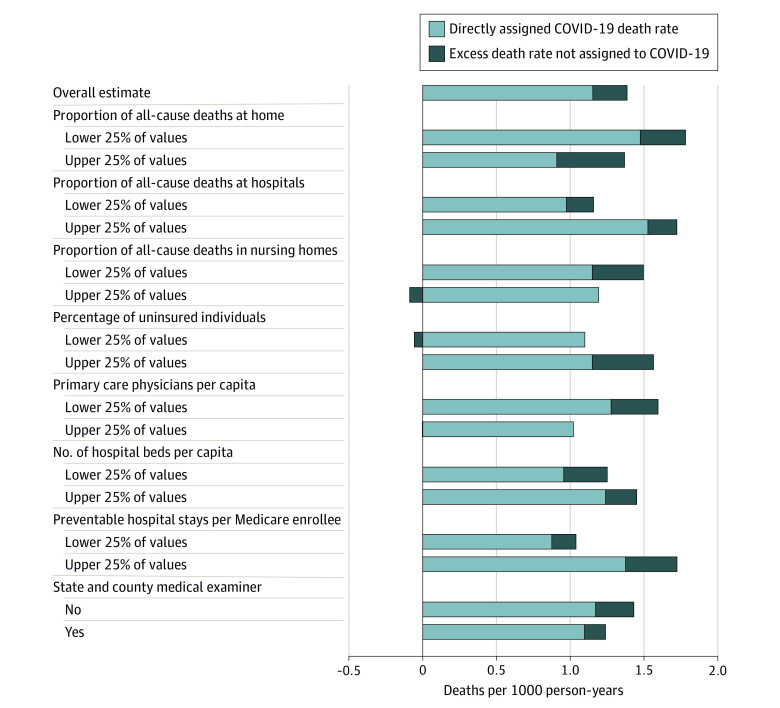
Decomposition of Excess Death Rates Across Strata of Health Care Factors in 2096 US Counties in 2020 Coefficients were generated according to the model given in the eMethods in the Supplement. The model was weighted by the 2020 population and fully stratified by health system factors. For continuous measures, factors were divided into population-weighted quartiles. Estimated death rates to the left of the observed death rate estimate indicate a negative prediction for the excess death rate not assigned to COVID-19 because β_2_ was less than 1.

This study included 2096 counties with 319.1 million residents, and 11.0% of the population was without health insurance. Figure 1 shows the percentage of excess deaths not assigned to COVID-19 across stratified models. The percentage of excess deaths not assigned to COVID-19 was higher in counties with more uninsured individuals (27%; 95% CI, 23%-30%) than in counties with fewer uninsured individuals (−5%; 95% CI, −14% to 3%). The percentage was also higher in counties with fewer primary care physicians per capita (20%; 95% CI, 16%-24%) than in counties with more primary care physicians (0%; 95% CI, −12% to 11%). The percentage was higher in counties in which more deaths at home (34%; 95% CI, 29%-38%) and fewer deaths in nursing homes (23%; 95% CI, 18%-28%) were reported than in counties in which fewer deaths at home (17%; 95% CI, 13%-21%) and more deaths in nursing homes (−8%; 95% CI, −18% to 2%) were reported. Figure 2 shows direct COVID-19 death rates and estimated excess death rates not assigned to COVID-19 in each stratum.

## Discussion

In this cross-sectional study, a greater proportion of excess deaths were not assigned to COVID-19 in counties with reduced access to health insurance and primary care and in counties with more at-home deaths. Reduced access to health care may prevent a patient from receiving COVID-19 testing and diagnosis, which may reduce the probability of valid cause-of-death assignment. Counties in which residents were more likely to die at home may have been places where indirect deaths, such as deaths from drug overdose, were more likely to have occurred; however, these factors were beyond the scope our study. Cause of death may also be less apparent for at-home deaths, and certifiers may have to make educated guesses based on a patient’s medical history.^5^ Dying at home may also be associated with an increased possibility of a coroner being involved in death certification. Coroners are often lay people who receive less professional training in death certification than medical examiners.^6^ Limitations of this analysis include use of provisional data; a lack of disaggregated data by age, sex, and race and ethnicity; and a lack of adjustment for other potential factors.

Regardless of the source of the discrepancy, our analysis suggests that marked variation in cause-of-death attribution occurred during the study period. Imprecise cause-of-death ascertainment may obscure the populations most at risk for COVID-19, leading to inadequate policy responses.

## Notes

Supplement.
eMethods.
