# Supplementary material for: Association of Health Care Factors With Excess Deaths Not Assigned to COVID-19 in the US
Source: JAMA Netw Open. 2021 Sep 13;4(9):e2125287. doi: 10.1001/jamanetworkopen.2021.25287 (PMC8438594; doi:10.1001/jamanetworkopen.2021.25287)
Supplement: Supplement. — eMethods. [file jamanetwopen-e2125287-s001.pdf]

Supplemental Online Content

Stokes AC, Lundberg DJ, Bor J, Elo IT, Hempstead K, Preston SH. Association of health care factors with excess deaths not assigned to COVID-19 in the US. *JAMA Netw Open*. 2021;4(9):e2125287. doi:10.1001/jamanetworkopen.2021.25287

eMethods.

This supplemental material has been provided by the authors to give readers additional information about their work.

© 2021 Stokes AC et al. *JAMA Network Open*.

eMethods.

Model Specification

We modeled all-cause mortality in 2020 as a function of historical all-cause mortality, directly assigned Covid-19 mortality in 2020, and an error term using Ordinary Least Squares regression with county units weighted by their estimated population in 2020:

$$M(i) = \alpha + \beta_1 M^*(i) + \beta_2 C(i) + \varepsilon, \text{ where}$$

$M(i)$ = Death rate from all causes in county i in 2020

$M^*(i)$ = Average death rate from all causes, county i in 2013-2018

$C(i)$ = Covid-19 death rate in county i in 2020

α = Changes in mortality that are independent of Covid-19 and common across regions

β_1 = Represents the extent to which past levels of all-cause mortality in a county are replicated in 2020

β_2 = Represents the extent to which mortality from Covid-19 affects all-cause mortality in 2020 after adjusting for historical mortality patterns

ε = Error term

Interpretation of Coefficients:

1. Combinations of α and β_1 indicate how mortality changes that are not associated with Covid-19 vary with the level of all-cause mortality in 2013-18. If β_1 = 1.0, this would indicate that all-cause mortality in 2020 was on average equal to that in 2013-18, plus or minus the value of α.

2. β_2 reflects the number of excess deaths that occurred for every 1 directly assigned Covid-19 death. For example, if β_2 were equal to 1.34, this means that for every 1 directly assigned Covid-19 death that occurred, 0.34 excess deaths occurred that were not assigned to Covid-19. If β_2 were less than 1, this would mean that Covid-19 is over-recorded as a cause of death or reductions in mortality occurred for other causes.

Stratification of the Model:

To examine whether characteristics at the county or state-level (health care factors) were associated with having a higher percentage of excess deaths not assigned to Covid-19, we fully stratified our model and compared the β_2 coefficient across strata. For continuous measures, factors were divided into population weighted quartiles, and we compared the upper and lower 25% of values.

© 2021 Stokes AC et al. *JAMA Network Open.*

Formulas

Formula for Calculating the Percent of Excess Deaths Not Assigned to Covid-19:

We calculated the percent of excess deaths not assigned to Covid-19 for each stratum using the β_2 coefficient produced by our stratified models. The formula was as follows:

Percent of Excess Deaths Not Assigned to Covid-19 = (β_2 Coefficient – 1) / β_2 Coefficient

For instance, in counties that had a higher percentage uninsured, the β_2 Coefficient was 1.36. This indicates that 1.36 excess deaths occurred for every 1 directly assigned Covid-19 deaths, suggesting that 0.36 out of 1.36 excess deaths were not assigned to Covid-19.

Percent of Excess Deaths Not Assigned to Covid-19 = (1.36 – 1) / 1.36 = 0.36 / 1.36 = 26%

This implies that 26% of excess deaths were not assigned to Covid-19 in these counties.

Formula for Predicting Excess Deaths Not Assigned to Covid-19:

We determined the average observed directly assigned Covid-19 death rate for each stratum by calculating the weighted mean of observed directly assigned Covid-19 mortality. We then predicted the excess death rate not assigned to Covid-19 using the β_2 coefficient as follows:

Excess Death Rate Not Assigned to Covid-19 = Mean Directly Assigned Covid-19 Rate • (β_2 Coefficient – 1)

© 2021 Stokes AC et al. *JAMA Network Open.*

Data Sources

Health Care Factors:

Variable:	Data Source:
% Uninsured	https://www.countyhealthrankings.org/explore-health-rankings/rankings-data-documentation
Primary Care Physicians per Capita	https://www.countyhealthrankings.org/explore-health-rankings/rankings-data-documentation
Number of Hospital Beds per Capita	https://carlsonschool.umn.edu/mili-misrc-covid19-tracking-project
Preventable Hospital Stays per Medicare Enrollee	https://www.countyhealthrankings.org/explore-health-rankings/rankings-data-documentation
% of All-Cause Deaths Occurring at Home, 2013-2018	https://wonder.cdc.gov/ucd-icd10.html
% of All-Cause Deaths Occurring in Inpatient Settings, 2013-2018	https://wonder.cdc.gov/ucd-icd10.html
% of All-Cause Deaths Occurring at Nursing Homes or Long-Term Care Facilities, 2013-2018	https://wonder.cdc.gov/ucd-icd10.html
Presence of a State & County Medical Examiner	https://www.cdc.gov/phlp/publications/coroner/death.html (Classifies states as having a state medical examiner or not) Data on coroners scraped from publicly available state/county sites

Mortality and Population Data:

Variable:	Data Source:
All-Cause and Covid-19 Deaths in a County from January 1 to December 31, 2020 (Reported by March 12, 2021)[a]	https://data.cdc.gov/NCHS/AH-County-of-Residence-Provisional-COVID-19-Deaths/75vb-d79q
All-Cause Deaths in a County from January 1 to December 31, 2013-2018	https://wonder.cdc.gov/ucd-icd10.html
County Population Estimates, 2013-2018	https://www.census.gov/data/datasets/time-series/demo/popest/2010s-counties-total.html?#
County Population Estimates, 2020	Special request from the U.S. Census Bureau

© 2021 Stokes AC et al. *JAMA Network Open.*

Sample Exclusion Criteria:

- Counties in the NCHS Database with Covid-19 and All-Cause Mortality Data for 2020 (n=3,140)
- Excluded Counties Missing All-Cause Mortality Data for 2013-2018 (n=2)
- Limited to Counties with 20+ Covid-19 Deaths (n=1,042)
- Final County Analytic Sample[b] (n=2,096)

a. To account for possible lags in mortality reporting, we used data generated on March 12, 2021 (ten weeks after the end date), meaning that all deaths occurring before December 31, 2020 that were reported before March 12, 2021 were captured. Prior analyses of NCHS provisional data have found that provisional mortality data can have low completeness in the first month after a death occurs but are more than 75% complete within 8 weeks after a death.
b. Counties had complete data for each of the characteristics we examined.

© 2021 Stokes AC et al. *JAMA Network Open*.
